# Overexpression of the scaffold WD40 protein WRAP53*β* enhances the repair of and cell survival from DNA double-strand breaks

**DOI:** 10.1038/cddis.2016.172

**Published:** 2016-06-16

**Authors:** H Rassoolzadeh, S Böhm, E Hedström, H Gad, T Helleday, S Henriksson, M Farnebo

**Affiliations:** 1 Cancer Centrum Karolinska, Department of Oncology-Pathology, Karolinska Institutet, Stockholm, Sweden; 2 Science for Life Laboratory, Division of Translational Medicine and Chemical Biology, Department of Medical Biochemistry and Biophysics, Karolinska Institutet, Stockholm, Sweden

## Abstract

Altered expression of the multifunctional protein WRAP53*β* (WD40 encoding RNA Antisense to p53), which targets repair factors to DNA double-strand breaks and factors involved in telomere elongation to Cajal bodies, is linked to carcinogenesis. While loss of WRAP53*β* function has been shown to disrupt processes regulated by this protein, the consequences of its overexpression remain unclear. Here we demonstrate that overexpression of WRAP53*β* disrupts the formation of and impairs the localization of coilin to Cajal bodies. At the same time, the function of this protein in the repair of DNA double-strand breaks is enhanced. Following irradiation, cells overexpressing WRAP53*β* exhibit more rapid clearance of phospho-histone H2AX (*γ*H2AX), and more efficient homologous recombination and non-homologous end-joining, in association with fewer DNA breaks. Moreover, in these cells the ubiquitylation of damaged chromatin, which is known to facilitate the recruitment of repair factors and subsequent repair, is elevated. Knockdown of the ubiquitin ligase involved, ring-finger protein 8 (RNF8), which is recruited to DNA breaks by WRAP53*β*, attenuated this effect, suggesting that overexpression of WRAP53*β* leads to more rapid repair, as well as improved cell survival, by enhancing RNF8-mediated ubiquitylation at DNA breaks. Our present findings indicate that WRAP53*β* and RNF8 are rate-limiting factors in the repair of DNA double-strand breaks and raise the possibility that upregulation of WRAP53*β* may contribute to genomic stability in and survival of cancer cells.

We previously identified the RNA produced from the *WRAP53* (WD40 encoding RNA Antisense to p53) gene as an antisense transcript (WRAP53*α*) that stabilizes the tumor suppressor p53.^1^ In addition, this gene encodes the WRAP53*β* protein (also referred to as WRAP53 or WDR79 or TCAB1), which does not regulate p53 but instead is involved in the regulation of telomere elongation and repair of DNA double-strand breaks by recruiting telomerase to nuclear Cajal bodies and the repair factor RNF8 to these break, respectively.^2, 3^ The role played by WRAP53*β* in the repair of DNA double-strand breaks is independent of p53, as WRAP53*β* regulates DNA repair also in cells that lack p53 expression.^3, 4^ WRAP53*β* also directs coilin, the survival of motor neuron (SMN) protein and small Cajal body-associated (sca) RNAs to Cajal bodies.^2, 5, 6^

Several lines of evidence indicate that WRAP53*β* itself also acts as a tumor suppressor. For example, mutations that attenuate its nuclear localization and telomere function cause dyskeratosis congenita, which enhances the risk for developing cancer.^7, 8^ These mutations also prevent binding to the DNA repair factor *γ*H2AX required for the accumulation of WRAP53*β* at DNA breaks,^9^ indicating that disturbed DNA repair may contribute to the pathogenesis of dyskeratosis congenita. Furthermore, loss of nuclear WRAP53*β* or single-nucleotide polymorphisms in the *WRAP53* gene is correlated with shorter survival of patients with head and neck, breast and ovarian cancer.^4, 10, 11, 12, 13, 14, 15^ In addition, attenuated expression of this protein correlates with disruption of the DNA damage response in ovarian tumors,^4^ as well as with resistance of head and neck cancer to radiotherapy.^14^ Accordingly, altered DNA repair may be the underlying cause of cancers associated with abnormalities in WRAP53*β* and influence the response of such tumors to treatment.

At the same time, overexpression of WRAP53*β* is observed in a variety of cancer cell lines compared with non-transformed cells.^16^ WRAP53*β* is also overexpressed in primary nasopharyngeal carcinoma,^17^ esophageal squamous cell carcinoma,^18^ non-small-cell lung cancer^19^ and rectal cancer^20^ and knockdown of this protein in cancer cells subsequently grafted into mice reduces the size of the tumors formed.^17, 19^ In esophageal squamous cell carcinoma, overexpression of WRAP53*β* was significantly correlated with tumor infiltration depth, clinical stage and lymph node metastasis.^18^ However, for none of the studies mentioned above significant associations between WRAP53*β* overexpression and patient survival were demonstrated. Therefore, although WRAP53*β* is clearly overexpressed in some tumor types, the clinical relevance of such overexpression remains unclear.

Thus, while loss of WRAP53*β* function impairs DNA repair and telomere maintenance, which enhances genomic instability and carcinogenesis, the role of WRAP53*β* overexpression in connection with carcinogenesis is poorly understood. Here, we examined the potential influence of such overexpression on the DNA damage response.

## Results

### Overexpression of WRAP53*β* disrupts Cajal bodies and the overexpressed protein is mainly soluble

**Figure 1:**
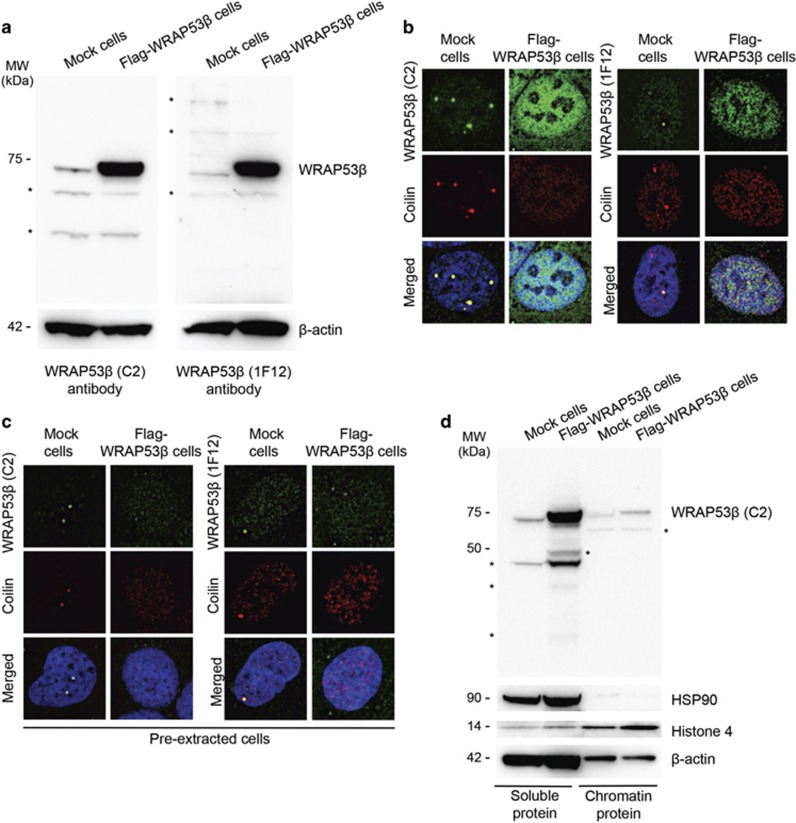
Overexpressed WRAP53*β* disrupts Cajal bodies and the overexpressed protein is mainly soluble. (**a**) Western blotting of the levels of WRAP53*β* in Mock (endogenous WRAP53*β*) or 1xFlag-WRAP53*β* (overexpressing WRAP53*β*) U2OS cells with WRAP53-C2 or WRAP53-1F12 antibodies. *β*-Actin was used as a loading control. Asterisk indicates unspecific bands. (**b**) Mock or Flag-WRAP53*β* U2OS cells were immunostained for WRAP53*β* (with WRAP53-C2 or WRAP53-1F12 antibodies) and coilin (a marker for Cajal bodies). In all immunofluorescent stainings, nuclei were stained with DAPI. (**c**) The soluble proteins in Mock and Flag-WRAP53*β* U2OS cells were removed by extraction before fixation with paraformaldehyde and immunostained for WRAP53*β* and coilin. (**d**) Western blotting of the soluble and chromatin proteins of Mock and Flag-WRAP53*β* U2OS cells. Equal volumes from each fraction were loaded onto the gels. HSP90 and histone 4 were employed as markers for the soluble and chromatin fractions, respectively. The slower migration of the chromatin proteins of WRAP53*β* on the SDS gel compared to its soluble counterpart may be due to additional modifications of this protein when bound to chromatin. * indicates unspecific bands or bands of unknown origin

The WRAP53*β* protein is highly enriched in Cajal bodies, and to examine whether this localization is altered upon overexpression, the total protein lysate from human U-2 osteosarcoma (U2OS) cancer cells that stably overexpress Flag-tagged WRAP53*β* was analyzed with both rabbit *α*-WRAP53-C2 antibody, which detects both the nuclear and cytoplasmic forms, and the mouse monoclonal *α*-WDR79 clone 1F12, which detects the nuclear variant, and is the only antibody that can be used to visualize this protein in repair foci.^3, 4^ Examination of western blots with either of these antibodies revealed elevated expression of WRAP53*β* (Figure 1a).

Immunostaining of WRAP53*β* and its interaction partner coilin (a marker for Cajal bodies) in control cells expressing endogenous WRAP53*β* revealed enrichment of both of these factors in Cajal bodies, as expected (Figure 1b).^5^ In contrast, no Cajal bodies were observed in the cells overexpressing WRAP53*β*, where this protein and coilin were distributed throughout the nucleoplasm (Figure 1b), in agreement with previous findings.^5^ Nor did reduction of background nucleoplasmic staining through pre-extraction of soluble proteins uncover any accumulation of WRAP53*β* or coilin in Cajal bodies in the cells overexpressing WRAP53*β*, although this treatment reduced total WRAP53*β* staining, potently indicating that most of the overexpressed protein is soluble (Figure 1c). Indeed, western blotting of the soluble and chromatin proteins of WRAP53*β*-overexpressing cells confirmed that most of the overexpressed WRAP53*β* was soluble, although the amount of this protein bound to chromatin also is increased (Figure 1d). Taken together, these findings demonstrate that overexpression of WRAP53*β* impairs accumulation of both this protein itself and coilin in Cajal bodies, with most of the overexpressed WRAP53*β* being in soluble form.

### Overexpression of WRAP53*β* does not influence its recruitment to sites of DNA damage

**Figure 2:**
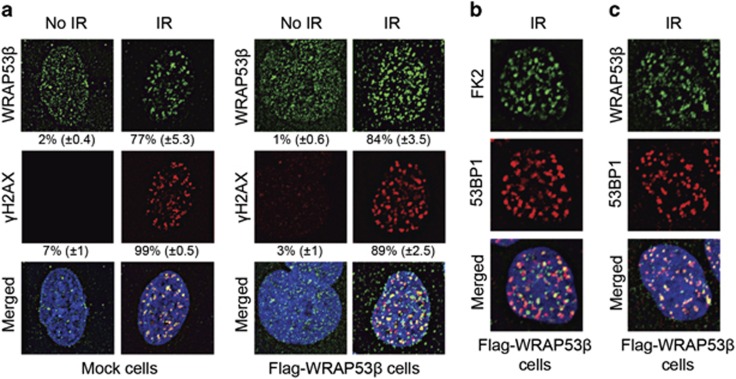
WRAP53*β* accumulates at DNA double-strand breaks in cells overexpressing this protein. (**a**) Mock or Flag-WRAP53*β* cells were left untreated or irradiated (IR) with 6 Gy, and 1 h later, their soluble proteins were removed by pre-extraction and the cells were then immunostained for WRAP53*β* (with WRAP53-1F12 antibody) and *γ*H2AX. The numbers represent the percentage of 100–200 cells counted whose nuclei contained >10 WRAP53*β* or *γ*H2AX foci. Means±S.D. are shown, *n*=3. (**b** and **c**) After irradiation and pre-extraction as above, Flag-WRAP53*β* cells were immunostained for the proteins indicated. The FK2 antibody recognizes conjugated ubiquitin

Next, we explored the localization of WRAP53*β* following exposure of cells to ionizing radiation (IR), which causes DNA double-strand breaks. WRAP53*β* localized rapidly to these breaks, to an extent of 84% in overexpressing cells in comparison with only 77% in the control cells (Figure 2a), which may simply reflect better visualization because of higher amounts of WRAP53*β* in the repair foci of the former. These foci also contained *γ*H2AX, a marker for sites of DNA damage known to interact with WRAP53*β* (Figure 2a).^9^

Ubiquitylation of damaged chromatin, a process regulated by the ubiquitin ligases RNF8 and RNF168 in conjunction with WRAP53*β*,^3^ has an important role in the downstream recruitment of repair factors.^3, 21, 22, 23, 24, 25^ Repair foci containing WRAP53*β* were also enriched in conjugated ubiquitin (detected using the FK2 (antibody clone recognizing K^29^-, K^48^- and K^63^-linked polyubiquitylated and monoubiquitylated protein) antibody) (Figure 2b), as well as in tumor protein p53 binding protein 1 (53BP1) (Figures 2b and c), a downstream repair factor that requires ubiquitin signaling for its recruitment to DNA breaks.^3^ Thus, we conclude that WRAP53*β*-mediated targeting of repair factors to DNA double-strand breaks is not disturbed in cells that overexpress WRAP53*β*.

### Repair of double-strand breaks in DNA is enhanced in cells that overexpress WRAP53*β*

**Figure 3:**
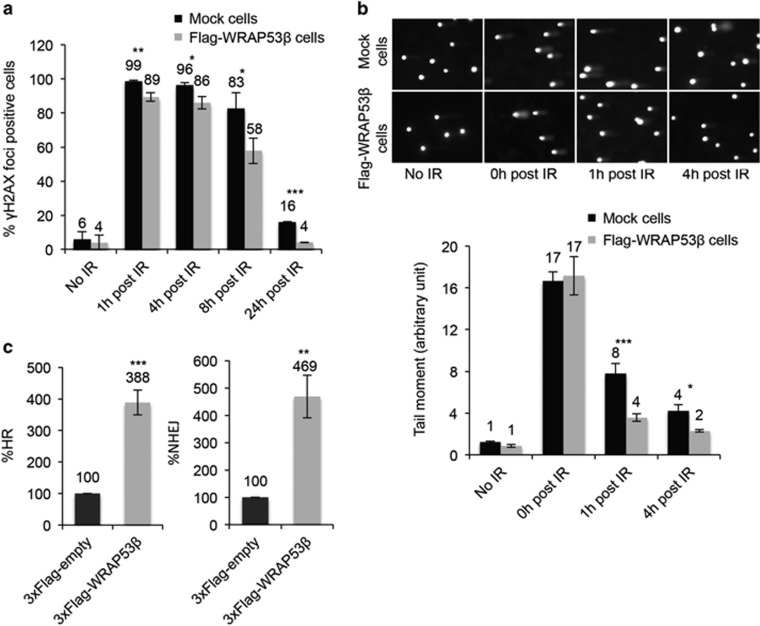
Overexpression of WRAP53*β* enhances the repair of double-strand breaks. (**a**) The percentage of 200 Mock and Flag-WRAP53*β* cells counted, irradiated with 6 Gy and then fixed at the time-points indicated, whose nuclei contained >10 *γ*H2AX foci. (**b**) Alkaline Comet assay of Mock and Flag-WRAP53*β* cells, either untreated (control) or exposed to 20 Gy IR, and then allowed to recover for 0, 1 or 4 h. The tail moment of 200 comets were analyzed for each experiment, *n*⩾3. (**c**) FACS analysis of HR and NHEJ efficiency following transient transfection of U2OS cells with the 3xFlag-Empty or 3xFlag-WRAP53*β* vectors for 48 h. In all cases, the values presented are means±S.D., *n*=3. *P<0.05, **P<0.01 and ***P<0.001, as determined by Student's *t*-test

Interestingly, in the cells overexpressing WRAP53*β* clearance of *γ*H2AX-containing foci formed in response to irradiation was more rapid than in control cells (Figure 3a), indicating faster DNA repair. Moreover, comet assays revealed that the cells overexpressing WRAP53*β* contained fewer DNA breaks both 1 and 4 h after irradiation (Figure 3b).

To examine whether more efficient DNA repair could explain these differences, we measured the efficiency of homologous recombination (HR) and non-homologous end-joining (NHEJ), repair pathways involved in the repair of double-strand breaks, using green fluorescent protein (GFP)-reporter assays.^26, 27^ In the case of HR, this assay involves U2OS cells carrying the direct repeat–GFP recombination construct, in which expression of exogenous I-*Sce*I introduces a single double-strand break and repair of this break by HR produces a functional GFP. The NHEJ assay works in a similar manner, except that the GFP reporter (EJ5-GFP) contains two I-*Sce*I sites, which upon cleavage generate two incompatible DNA ends repaired by NHEJ. Strikingly, transient overexpression of Flag-WRAP53*β* enhanced the efficiency of HR and NHEJ by ~4-fold (Figure 3c). Thus, we conclude that in cells overexpressing WRAP53*β*, HR and NHEJ are more efficient and consequently repair of DNA breaks more rapid.

### Overexpression of WRAP53*β* enhances DNA repair by promoting RNF8-mediated ubiquitylation of histones

**Figure 4:**
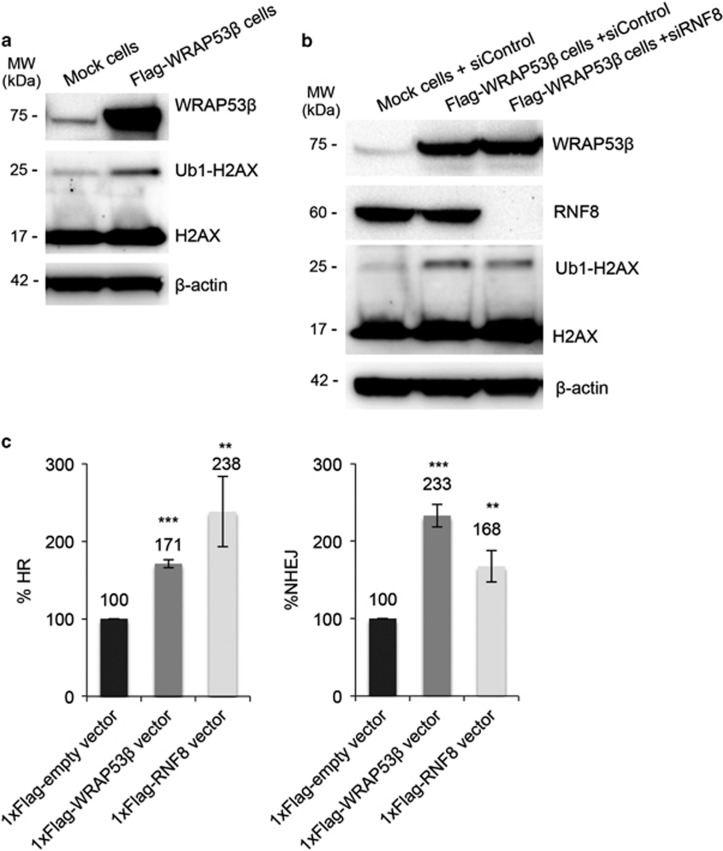
Overexpression of WRAP53*β* enhances histone ubiquitylation by RNF8. (**a**) Mock and Flag-WRAP53*β* cells were irradiated with 6 Gy and then allowed to recover for 1 h, after which western blotting for WRAP53*β*, H2AX and *β*-actin was performed. (**b**) Mock or Flag-WRAP53*β* cells were treated with the siRNAs indicated for 48 h, then irradiated with 6 Gy and allowed to recover for 1 h, after which western blotting for WRAP53*β*, RNF8, H2AX and *β*-actin was performed. (**c**) FACS analysis of HR and NHEJ efficiency following transient transfection of U2OS cells with the 1xFlag-Empty, 1xFlag-WRAP53*β* or 1xFlag-RNF8 vectors for 48 h. The values presented are means±S.D., *n*=3. **P<0.01 and ***P<0.001, as determined by Student's *t*-test

WRAP53*β* orchestrates repair of DNA double-strand breaks by recruiting the critical ubiquitin ligase RNF8 to DNA lesions.^3^ Ubiquitylation of histone H2AX, a known target of RNF8 at sites of DNA damage, was clearly elevated in cells overexpressing WRAP53*β* following irradiation (Figure 4a) and knockdown of RNF8 attenuated this effect (Figure 4b), suggesting that overexpression of WRAP53*β* stimulates RNF8-regulated ubiquitylation of damaged chromatin. In agreement with this idea, overexpression of RNF8 itself enhanced the efficiency of both HR and NHEJ repair to an extent similar to overexpression of WRAP53*β* (Figure 4c). Altogether, these findings indicate that overexpression of WRAP53*β* facilitates repair of DNA breaks by promoting RNF8-mediated ubiquitylation of associated histones, a process important for the assembly of downstream repair factors.

### Overexpression of WRAP53*β* promotes cells' survival following DNA damage

**Figure 5:**
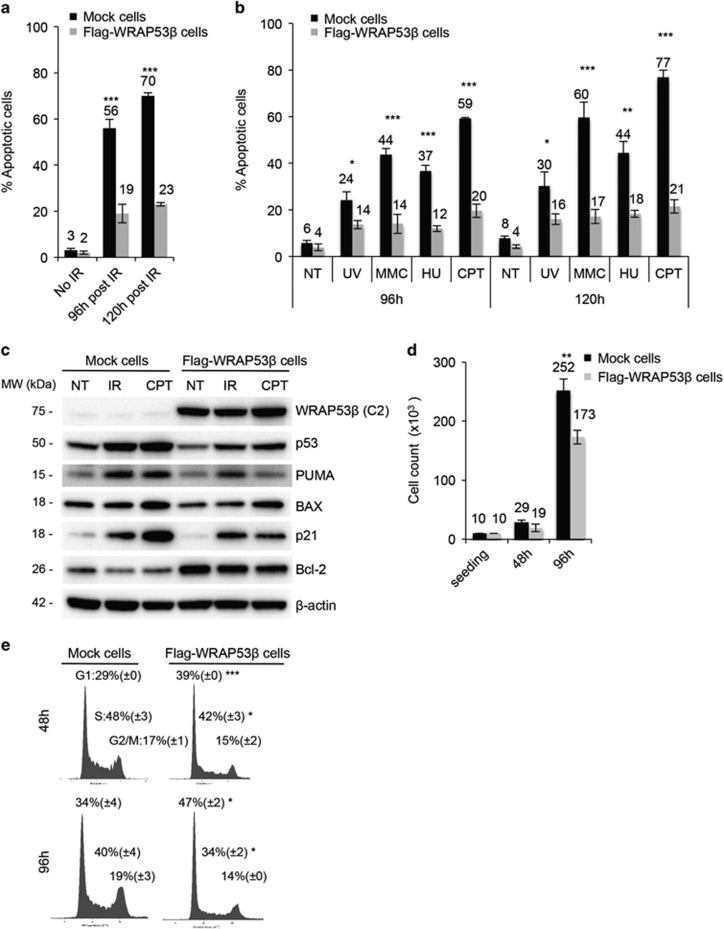
Cells that overexpress WRAP53*β* are less sensitive to apoptosis induced by DNA damage. (**a**) Mock and Flag-WRAP53*β* cells were irradiated (10 Gy), harvested 96 or 120 h later and analyzed by FACS for positive staining with Annexin V-PI, an indicator of apoptosis. (**b**) Mock and Flag-WRAP53*β* cells were treated with UV (30 J/m^2^), MMC (1 *μ*g/ml), HU (500 *μ*M), CPT (50 nM) or nothing (NT control) for 96 or 120 h, harvested and analyzed for Annexin V-PI positivity by FACS. (**c**) Mock and Flag-WRAP53*β* cells were left untreated (NT), irradiated (10 Gy) or treated with CPT (50 nM), harvested 72 h later and analyzed by western blotting for indicated proteins. (**d**) A total of 10 000 cells per ml of Mock and Flag-WRAP53*β* cells were seeded in 6-well plates, grown for 48 or 96 h, harvested and counted. The values presented are cells per ml. (**e**) Mock and Flag-WRAP53*β* cells were seeded, grown for 48 or 96 h, harvested, stained with PI and subjected to flow cytometry. The numbers indicate the % of cells in each cell cycle phase. Asterisk indicates the significance between the values in Flag-WRAP53*β* cells compared to the corresponding value in control cells. Percentages of sub-G1 and super-G2 are not shown. In all cases, the values presented are means±S.D., *n*=3. *P<0.05, **P<0.01 and ***P<0.001, as determined by Student's *t*-test

Tumors exhibiting altered responses to DNA damage can be either hypersensitive or resistant to genotoxic drugs. Interestingly, 70% of the control cells, but only 23% of those overexpressing WRAP53*β*, were apoptotic 5 days after irradiation (Figure 5a). The latter were also less sensitive to other genotoxic agents, including ultraviolet (UV) rays (42–47% reduction in the extent of apoptosis), mitomycin C (MMC; 68–72% decrease), hydroxyurea (HU; 59–68% decrease) and camptothecin (CPT; 66–73% decrease) (Figure 5b).

Examination of the apoptotic response following DNA damage in these cells revealed reduced proapoptotic signaling (p53, PUMA, BAX) in cells overexpressing WRAP53*β* compared with control cells and upregulation of the antiapoptotic protein Bcl-2 in the former (Figure 5c), indicating that an altered apoptotic program may contribute to the resistance of WRAP53*β*-overexpressing cells to genotoxic agents.

Furthermore, we examined the growth rate of these cells, as DNA-damaging drugs most effectively eliminate rapidly dividing cells, while quiescent or slow-cycling cells are less susceptible.^28^ Interestingly, cells overexpressing WRAP53*β* grow slower than control cells (Figure 5d). The lower amount of cells overexpressing WRAP53*β* at 48 or 96 h after seeding compared with control cells was not because of apoptosis (Figure 5b – NT fraction) or cell cycle arrest (Figure 5e). However, the cell cycle distribution was slightly different between cells overexpressing WRAP53*β* and control cells, with more cells in the G1 phase and less in the S phase for the former, whereas the amount of cells in the G2 phase was the same for both cell lines. Taken together, our results demonstrate that overexpression of WRAP53*β* results in faster DNA repair, an altered apoptotic response and slower proliferation/cycling, thereby promoting cell survival following DNA damage.

## Discussion

Although high levels of WRAP53*β*, a scaffold protein involved in the intracellular trafficking of factors to Cajal bodies and DNA breaks,^3, 5, 29^ are present in various cancers,^16, 17, 18, 19, 20^ it remains unclear how such overexpression may alter its functions. Here we report that overexpression of WRAP53*β* disturbs its recruitment and maintenance of factors to Cajal bodies, while at the same time enhancing the repair of DNA double-strand breaks. In cancer cells stably overexpressing this protein, WRAP53*β* was mislocalized in the nucleoplasm, in agreement with previous findings.^5^ Coilin, a marker protein for the Cajal body, demonstrated similar mislocalization, and thus no Cajal bodies appeared to have been formed. It is important to note that the overexpression in our cells was relatively high and that cells overexpressing WRAP53*β* at lower levels are still capable of forming Cajal bodies.^5^ Moreover, these cells only overexpress the open-reading frame of the protein and not the sequence of the WRAP53*α* transcript involved in regulating p53.

Intriguingly, in contrast to its absence from Cajal bodies, WRAP53*β* still localized to sites of DNA damage sites in overexpressing cells. Most of the overexpressed WRAP53*β* was soluble and may have competed with endogenous soluble WRAP53*β* for factors important for localization of this protein and associated factors. If soluble WRAP53*β* controls targeting of factors to Cajal bodies and the chromatin-bound fraction participates in DNA repair, this could explain why only its function related to the Cajal body are disrupted by overexpression.

Indeed, WRAP53*β* translocates certain components, including the SMN complex, from the cytoplasm to Cajal bodies,^5^ suggesting the involvement of a more mobile form of WRAP53*β*. Moreover, Cajal bodies alternate between passive diffusion within the nucleoplasm and transient immobilization through association with chromatin. In contrast, WRAP53*β* is recruited to sites of DNA damage from the nuclear pool alone, that is, no cytoplasmic shuttling is involved.^4^ In addition, many repair factors are bound to chromatin even before damage, allowing rapid redistribution to damaged sites when needed.

Our present observations also show that overexpression of WRAP53*β* enables more rapid repair of DNA double-strand breaks by HR and NHEJ. An open chromatin configuration allows more efficient repair of DNA double-strand breaks, providing greater resistance to such damage.^30^ Ubiquitylation of damaged chromatin is rate limiting for the accumulation of downstream repair factors,^3, 21, 22, 23, 24^ and, indeed, we found that overexpression of WRAP53*β* elevated ubiquitylation of H2AX. Knockdown of the ubiquitin ligase involved, RNF8, abrogated this effect in agreement with our previous report that WRAP53*β* targets RNF8 to DNA breaks.^3^

Rapid exchange of RNF8 at sites of DNA damage and the small number of copies of this protein in repair foci have been proposed to reflect its rate-limiting role in repair.^31^ Overexpression of WRAP53*β* may increase the level of RNF8 in repair foci by tethering it to damaged chromatin, thus slowing down its otherwise rapid turnover and promoting ubiquitylation. Our finding that overexpression of RNF8 itself enhanced HR and NHEJ repair supports the suggestion that elevating its local concentration may boost its action at DNA breaks. Moreover, RNF8 promotes decondensation of damaged chromatin by recruiting the chromatin-remodeling factor CHD4 (chromodomain helicase DNA-binding 4)^32^ and it is possible that WRAP53*β* aids in this decondensation, thereby enhancing chromatin accessibility to DNA repair. However, further investigation of the potential role of WRAP53*β* in the organization of chromatin is required.

Overexpression of WRAP53*β* also enhances cells survival following exposure to radiation and other DNA-damaging agents. We showed earlier that knockdown of WRAP53*β* in cancer cell lines, including the U2OS cells examined here, triggers mitochondria-dependent apoptosis and that overexpression of Bcl-2 protects against this apoptosis.^16^ Thus, Bcl-2 and/or other antiapoptotic proteins may be upregulated in cells that overexpress WRAP53*β*, protecting them from apoptosis. Indeed, we observed higher expression of Bcl-2 in these cells as well as diminished proapoptotic signaling. Moreover, we reveal that cells overexpressing WRAP53*β* proliferate significantly slower than cells with endogenous expression of this protein. Thus, it appears like cells overexpressing WRAP53*β* have acquired multiple resistance mechanisms, including increased DNA repair, overexpression of the antiapoptotic protein Bcl-2 and slow cycling, rendering them less sensitive to initiation of apoptosis by DNA damage.

The contradictory findings concerning the contribution of WRAP53*β* to cancer may involve timing. Inactivation could help initiate tumor development by impairing DNA repair and telomere maintenance and causing genomic instability. Indeed, inactivation of WRAP53*β* as a result of inherited mutations leads to the cancer susceptibility-disorder dyskeratosis congenita.^7^ In contrast, overexpression could be a late event in tumor progression, enhancing DNA repair in transformed cells suffering from replication stress and thereby promoting their maintenance and survival. Alternatively, high expression of WRAP53*β* observed in primary tumors could reflect accumulation of misfolded inactive WRAP53*β*, as mutations in this protein disrupt its folding by the chaperonin TRiC, which results in cytoplasmic accumulation and lost nuclear activity of mutated WRAP53*β*.^9, 33^ Similarly, p53 was initially believed to be an oncogene because of its overexpression in cancer, but later on found to be a tumor suppressor frequently inactivated in cancer, where the mutations cause overexpression of inactive p53 owing to reduced degradation of the protein.^34^ In any case, understanding the function of WRAP53*β* in health and diseases, such as cancer, may open novel therapeutic strategies.

## Materials and Methods

### Cells and culture conditions

Mock and Flag-WRAP53*β* U2OS cells were maintained in McCoy's 5A medium (HyClone, Thermo Scientific, Stockholm, Sweden), selected with 10 *μ*g/ml blasticidine S (InvivoGen), and DR-GFP and EJ5-GFP U2OS cells were maintained in high glucose DMEM (HyClone), supplemented with 10% fetal bovine serum (Hyclone) and 2.5 *μ*g/ml plasmocin (InvivoGen) at 37 °C in 5% CO_2_ humidified chamber. Flag-WRAP53*β* cells overexpress the open-reading frame of the protein tagged with 1xFlag.

### Antibodies

#### Primary

Rabbit *α*-WRAP53-C2 (cat. no. PA-2020-100; Innovagen AB, Lund, Sweden), mouse monoclonal *α*-WDR79 (clone 1F12, cat. no. H00055135-M04; Abnova, Stockholm, Sweden), mouse *α*-coilin (cat. no. sc-56298; Santa Cruz Biotechnology, Heidelberg, Germany), rabbit *α*-coilin (cat. no. sc-32860; Santa Cruz Biotechnology), mouse *α*-*γ*H2AX (cat. no. 05-636; Millipore, Solna, Sweden), rabbit *α*-*γ*H2AX (cat. no. 2577; Cell Signaling, Danvers, MA, USA), mouse *α*-conjugated ubiquitin (FK2) (cat. no. ST1200; Calbiochem, Millipore), rabbit *α*-53BP1 (cat. no. NB100-904; Novus Biologicals), rabbit *α*-H2AX (cat. no. ab11175; Abcam), mouse *α*-RNF8 (cat. no. sc-271462; Santa Cruz Biotechnology), rabbit *α*-H4 (cat. no. ab10158; Abcam), mouse *α*-p53 (cat. no. sc-126; Santa Cruz), rabbit *α*-PUMA (cat. no. ab9643; Abcam), rabbit *α*-BAX (cat. no. ab32503; Abcam), mouse *α*-Bcl-2 (cat. no. M0887; Dako), rabbit *α*-p21 (cat. no. ab109199; Abcam) and mouse heat-shock protein 90 (HSP90) *α*/β (cat. no. sc-13119; Santa Cruz Biotechnology).

#### Secondary

Goat *α*-rabbit HRP (cat. no. 7074; Cell Signaling), horse *α*-mouse HRP (cat. no. 7076; Cell Signaling), goat *α*-rabbit Alexa Fluor 488 (cat. no. A11008; Invitrogen, Thermo Scientific), goat *α*-mouse Alexa Fluor 488 (cat. no. A11029, Invitrogen, Thermo Scientific), donkey *α*-rabbit Alexa Flour 594 (cat. no. A21207; Invitrogen) and donkey *α*-mouse Alexa Fluor 594 (cat. no. A21203; Invitrogen).

### Immunofluorescence microscopy

Cells grown on sterilized coverslips were fixed with 4% paraformaldehyde for 15 min at room temperature. They were then permeabilized with 0.1% Triton X-100 for 5 min at room temperature, followed by 30 min of blocking in blocking buffer (2% bovine serum albumin (BSA), 5% glycerol, 0.2% Tween-20, 0.1% NaN_3_). The coverslips were subsequently incubated for 1 h in primary antibody, followed by 40 min in secondary antibody, both diluted in blocking buffer, and finally mounted with Vectashield mounting medium containing DAPI (4′,6-diamidino-2-phenylindole; Vector Laboratories, Burlingame, CA, USA). Images were acquired with an LSM700 confocal microscope (Zeiss, Stockholm, Sweden), mounted on Axio observer.Z1 (Zeiss) equipped with Plan-Apochromat x63/1.4 oil immersion lens and processed with using Zen 2012 Black (Zeiss).

#### Pre-extraction

The cells were first washed with PBS and then incubated for 3 min at room temperature with cytoskeleton buffer (CSK) (10 mM Pipes, pH 7.0, 100 mM NaCl, 300 mM sucrose, 3 mM MgCl_2_ and 0.7% Triton X-100) and thereafter for another 3 min with the same CSK buffer supplemented with 0.3 mg/ml RNase A (CSK+R). Following these treatments, the cells were washed once again with PBS and then fixed in 4% paraformaldehyde.

### Western blotting

Cells were harvested, washed and lysed in ice-cold lysis buffer (100 mM Tris-HCl, pH 8, 150 mM NaCl, 1% NP-40, 1% PMSF, 1% protease inhibitor cocktail) for 30 min on ice followed by sonication. The lysates obtained were centrifuged at 14 000 r.p.m. for 15 min at 4 °C and their protein concentrations determined with the Bradford assay (Bio-Rad). Thereafter, western blotting was performed by standard procedures.

### Cell fractionation

Cells were harvested, washed in PBS and lysed in low salt buffer (10 mM HEPES, pH 7.4, 10 mM KCl, 0.05% Nonidet P-40, 1 mM DTT, 1% protease inhibitor cocktail) for 5 min on ice, followed by centrifugation at 3600 × *g* for 5 min at 4 °C. The supernatants were centrifuged again at 13 000 × *g* for 10 min at 4 °C. The supernatant (soluble proteins) was transferred to a new Eppendorf tube. The pellet from the first centrifugation were washed two times in low salt buffer with centrifugation at 3000 × *g* for 5 min at 4 °C between each wash. The remaining pellet were lysed in nuclease buffer supplemented with micrococcal nuclease (150 nM NaCl, 5 mM MgCl_2_, 1x micrococcal nuclease buffer, BSA, 2000 gel units micrococcal nuclease (cat. no. M0247S; New England Biolabs, Ipswich, MA, USA) for 10 min at 37 °C, followed by centrifugation at 13 000 × *g* for 10 min at 4 °C and transferred to a new Eppendorf tube (chromatin proteins). The soluble and chromatin fractions were adjusted to equal volumes. In Figure 1d, equal volumes of each fraction were loaded on an SDS-PAGE gel, corresponding to ~50 *μ*g of soluble proteins and 3 *μ*g of chromatin proteins.

### Ionizing radiation

*γ*-Irradiation was performed with a ^137^Cs source (Scanditronix, Uppsala, Sweden) at the Karolinska Institutet (Stockholm, Sweden), at a photon dose rate of 0.5 Gy/min. Dosimetry was carried out with an ionization chamber as well as with ferro sulfate.

### Comet assay

The alkaline Comet assay was performed using the method described earlier with some modifications.^35^ Briefly, cells were irradiated with 20 Gy. After 0, 1 or 4 h, cells were harvested with trypsin and washed. A total of 150 000 cells were diluted in 300 *μ*l PBS, of which 100 *μ*l were mixed with 500 *μ*l low-melting-point agarose (1.2% (w/v) in PBS) to a final concentration of 1%. Of the resulting suspension, 100 *μ*l was layed on the top of a previously prepared normal-melting-point agarose (1% (w/v) in PBS) on fully-frosted slides and the suspension was covered with a 22x22 mm^2^ coverslip. The slides were put on ice for 10 min and then immersed in lysis buffer (2.5 M NaCl, 100 mM sodium-EDTA, 10 mM Tri-HCl, pH 10, 10% DMSO, 1% Triton X-100) for overnight at 4 °C in the dark. After lysis, slides were placed in alkaline electrophoresis buffer (0.3 M NaOH, 1 mM sodium-EDTA) for 30 min at room temperature to denature DNA and express alkali-labile sites. Electrophoresis was carried out at room temperature for 30 min at 25 V, 300 mA in a Comet Assay Tank (Thistle Scientific, Glasgow, UK). The slides were then washed two times in neutralizing buffer (0.4 M Tris-HCl, pH 7.4) for 30 min. DNA was stained with 40 *μ*l CybrGOLD diluted 1 : 1000 (Thermo Scientific). Two slides were run and analyzed for each experiment. On each slide, 100 comets were quantified at × 10 magnification using the Comet Assay IV Image analysis system (Perceptive Instruments, Bury Saint Edmunds, UK) in live-video mode on a Zeiss Axiovert 35 fluorescent microscope (Zeiss). Tail moment (arbitrary unit) is defined as the product of the tail length and the fraction of total DNA in the tail. Images were acquired using the same software. For the statistical analysis, a two-way ANOVA analysis was performed in GraphPad Prism software (GraphPad Software, La Jolla, CA, USA).

### Drug treatment

In all, 1 *μ*g/ml MMC (cat. no. 10107409001; Roche, Stockholm, Sweden), 500 *μ*M HU (cat. no. H8627; Sigma-Aldrich), 50 nM CPT (cat. no. C9911; Sigma-Aldrich, Stockholm, Sweden) were added to cells for 96 or 120 h.

### FACS analysis

For the analysis of apoptosis, cells were exposed to genotoxic agents and harvested at the indicated time-points with trypsin. Next, the samples were incubated with incubation buffer (10 mM HEPES (pH 7.4), 140 mM NaCl, 2.5 mM CaCl_2_) supplemented with Annexin-V-FLUOS (cat. no. 11828681001; Roche) and 0.1% propidium Iodide (PI) solution (cat. no. P4864; Sigma-Aldrich) for 15 min at room temperature. The cells were then dissolved in incubation buffer and analyzed for active Annexin V-PI staining by flow cytometry on a FACS Calibur (Becton Dickinson, Erembodegem, Belgium) using the Cell Quest software (Becton Dickinson). The sum of the Annexin V^+^-P^−^ (early apoptosis) and Annexin V^+^-PI^+^ (late apoptosis) population of cells were accounted as apoptotic cells.

For the analysis of the cell cycle, cells were seeded and 48 or 96 h later harvested with trypsin, washed in PBS and fixed in 60% ethanol overnight at 4 °C. After removal of the ethanol, the samples were incubated with a solution of RNase A/PI for 30 min at 37 °C and subsequently analyzed with a NovoCyte apparatus (ACEA Biosciences Inc., San Diego, CA, USA).

### HR and NHEJ assays

A total of 300 000 cells were seeded into 6-well plates. After 24 h, cells were transfected with an I-SceI vector together with a vector expressing Flag-Empty, Flag-WRAP53*β* or Flag-RNF8 using Lipofectamine 2000 (Invitrogen). 1xFlag vector corresponds to pCMV-Tag2 vector (Invitrogen) and 3xFlag vector to p3xFlag-CMV-9 (Sigma-Aldrich). The next day, media were changed, and 24 h after this, cells were harvested by trypsination, washed with PBS and the GFP signal arising from the recombination event was measured by flow cytometry on a FACS Calibur (as described above), with fluorescence detected in the FL1-H channel (logarithmic scale). The frequency of repair in cells transfected with the various plasmids was calculated relative to cells transfected with the empty plasmid. Each data point represents the mean±S.D. from three independent experiments.

### Cell count analysis

Cells were seeded and 48 or 96 h later harvested and counted using a Bürker chamber Brand (Sigma-Aldrich).

### siRNA transfections

Ten nanomoles of small interference RNA (siRNA), siRNF8 (cat. no. L-006900-00-0005; Dharmacon, Karlskoga, Sweden) or siControl (cat. no. 1027280, Qiagen), was transfected into cells using HiPerfect (Qiagen, Sollentuna, Sweden) transfection reagent in accordance with the supplier's recommendations.

## Abbreviations

WRAP53: WD40 encoding RNA antisense to p53
HR: homologous recombination
NHEJ: non-homologous end-joining
IR: ionizing irradiation
DAPI: 4′,6-diamidino-2-phenylindole
CSK: cytoskeleton buffer
siRNA: small interference RNA
*γ*H2AX: phospho-histone H2AX
RNF8: ring-finger protein 8
53BP1: tumor protein p53 binding protein 1
FK2: antibody clone recognizing K^29^-, K^48^- and K^63^-linked polyubiquitylated and monoubiquitylated protein
SMN: survival of motor neuron
UV: ultraviolet
MMC: mitomycin C
HU: hydroxyurea
CPT: camptothecin
PI: propidium iodide
HSP90: heat-shock protein 90
U2OS: U-2 osteosarcoma
BSA: bovine serum albumin

## References

[bib1] Mahmoudi S, Henriksson S, Corcoran M, Mendez-Vidal C, Wiman KG, Farnebo M. Wrap53, a natural p53 antisense transcript required for p53 induction upon DNA damage. Mol Cell 2009; 33: 462–471. 19250907 10.1016/j.molcel.2009.01.028

[bib2] Venteicher AS, Abreu EB, Meng Z, McCann KE, Terns RM, Veenstra TD et al. A human telomerase holoenzyme protein required for Cajal body localization and telomere synthesis. Science 2009; 323: 644–648. 19179534 10.1126/science.1165357 PMC2728071

[bib3] Henriksson S, Rassoolzadeh H, Hedstrom E, Coucoravas C, Julner A, Goldstein M et al. The scaffold protein WRAP53beta orchestrates the ubiquitin response critical for DNA double-strand break repair. Genes Dev 2014; 28: 2726–2738. 25512560 10.1101/gad.246546.114 PMC4265676

[bib4] Hedstrom E, Pederiva C, Farnebo J, Nodin B, Jirstrom K, Brennan DJ et al. Downregulation of the cancer susceptibility protein WRAP53beta in epithelial ovarian cancer leads to defective DNA repair and poor clinical outcome. Cell Death Dis 2015; 6: e1892. 26426684 10.1038/cddis.2015.250 PMC4632285

[bib5] Mahmoudi S, Henriksson S, Weibrecht I, Smith S, Soderberg O, Stromblad S et al. WRAP53 is essential for Cajal body formation and for targeting the survival of motor neuron complex to Cajal bodies. PLoS Biol 2010; 8: e1000521. 21072240 10.1371/journal.pbio.1000521 PMC2970535

[bib6] Tycowski KT, Shu MD, Kukoyi A, Steitz JA. A conserved WD40 protein binds the Cajal body localization signal of scaRNP particles. Mol Cell 2009; 34: 47–57. 19285445 10.1016/j.molcel.2009.02.020 PMC2700737

[bib7] Zhong F, Savage SA, Shkreli M, Giri N, Jessop L, Myers T et al. Disruption of telomerase trafficking by TCAB1 mutation causes dyskeratosis congenita. Genes Dev 2011; 25: 11–16. 21205863 10.1101/gad.2006411 PMC3012932

[bib8] Zhong FL, Batista LF, Freund A, Pech MF, Venteicher AS, Artandi SE. TPP1 OB-fold domain controls telomere maintenance by recruiting telomerase to chromosome ends. Cell 2012; 150: 481–494. 22863003 10.1016/j.cell.2012.07.012 PMC3516183

[bib9] Rassoolzadeh H, Coucoravas C, Farnebo M. The proximity ligation assay reveals that at DNA double-strand breaks WRAP53beta associates with gammaH2AX and controls interactions between RNF8 and MDC1. Nucleus 2015; 6: 417–424. 26734725 10.1080/19491034.2015.1106675 PMC4915514

[bib10] Garcia-Closas M, Kristensen V, Langerod A, Qi Y, Yeager M, Burdett L et al. Common genetic variation in TP53 and its flanking genes, WDR79 and ATP1B2, and susceptibility to breast cancer. Int J Cancer 2007; 121: 2532–2538. 17683073 10.1002/ijc.22985

[bib11] Medrek K, Magnowski P, Masojc B, Chudecka-Glaz A, Torbe B, Menkiszak J et al. Association of common WRAP 53 variant with ovarian cancer risk in the Polish population. Mol Biol Rep 2013; 40: 2145–2147. 23192612 10.1007/s11033-012-2273-9 PMC3563948

[bib12] Schildkraut JM, Goode EL, Clyde MA, Iversen ES, Moorman PG, Berchuck A et al. Single nucleotide polymorphisms in the TP53 region and susceptibility to invasive epithelial ovarian cancer. Cancer Res 2009; 69: 2349–2357. 19276375 10.1158/0008-5472.CAN-08-2902 PMC2666150

[bib13] Lan Q, Zhang L, Shen M, Jo WJ, Vermeulen R, Li G et al. Large-scale evaluation of candidate genes identifies associations between DNA repair and genomic maintenance and development of benzene hematotoxicity. Carcinogenesis 2009; 30: 50–58. 18978339 10.1093/carcin/bgn249 PMC2639030

[bib14] Garvin S, Tiefenbock K, Farnebo L, Thunell LK, Farnebo M, Roberg K. Nuclear expression of WRAP53beta is associated with a positive response to radiotherapy and improved overall survival in patients with head and neck squamous cell carcinoma. Oral Oncol 2015; 51: 24–30. 25456005 10.1016/j.oraloncology.2014.10.003

[bib15] Silwal-Pandit L, Russnes H, Borgen E, Skarpeteig V, Moen Vollan HK, Schlichting E et al. The sub-cellular localization of WRAP53 has prognostic impact in breast cancer. PLoS One 2015; 10: e0139965. 26460974 10.1371/journal.pone.0139965 PMC4603798

[bib16] Mahmoudi S, Henriksson S, Farnebo L, Roberg K, Farnebo M. WRAP53 promotes cancer cell survival and is a potential target for cancer therapy. Cell Death Dis 2011; 2: e114. 21368886 10.1038/cddis.2010.90 PMC3077286

[bib17] Sun CK, Luo XB, Gou YP, Hu L, Wang K, Li C et al. TCAB1: a potential target for diagnosis and therapy of head and neck carcinomas. Mol Cancer 2014; 13: 180. 25070141 10.1186/1476-4598-13-180 PMC4118648

[bib18] Rao X, Huang D, Sui X, Liu G, Song X, Xie J et al. Overexpression of WRAP53 is associated with development and progression of esophageal squamous cell carcinoma. PLoS One 2014; 9: e91670. 24626331 10.1371/journal.pone.0091670 PMC3953598

[bib19] Sun Y, Yang C, Chen J, Song X, Li Z, Duan M et al. Overexpression of WDR79 in non-small cell lung cancer is linked to tumour progression. J Cell Mol Med 2016; 20: 698–709. 26849396 10.1111/jcmm.12759 PMC5125931

[bib20] Zhang H, Wang DW, Adell G, Sun XF. WRAP53 is an independent prognostic factor in rectal cancer – a study of Swedish clinical trial of preoperative radiotherapy in rectal cancer patients. BMC Cancer 2012; 12: 294. 22805008 10.1186/1471-2407-12-294 PMC3504514

[bib21] Kolas NK, Chapman JR, Nakada S, Ylanko J, Chahwan R, Sweeney FD et al. Orchestration of the DNA-damage response by the RNF8 ubiquitin ligase. Science 2007; 318: 1637–1640. 18006705 10.1126/science.1150034 PMC2430610

[bib22] Marteijn JA, Bekker-Jensen S, Mailand N, Lans H, Schwertman P, Gourdin AM et al. Nucleotide excision repair-induced H2A ubiquitination is dependent on MDC1 and RNF8 and reveals a universal DNA damage response. J Cell Biol 2009; 186: 835–847. 19797077 10.1083/jcb.200902150 PMC2753161

[bib23] Huen MS, Grant R, Manke I, Minn K, Yu X, Yaffe MB et al. RNF8 transduces the DNA-damage signal via histone ubiquitylation and checkpoint protein assembly. Cell 2007; 131: 901–914. 18001825 10.1016/j.cell.2007.09.041 PMC2149842

[bib24] Mailand N, Bekker-Jensen S, Faustrup H, Melander F, Bartek J, Lukas C et al. RNF8 ubiquitylates histones at DNA double-strand breaks and promotes assembly of repair proteins. Cell 2007; 131: 887–900. 18001824 10.1016/j.cell.2007.09.040

[bib25] Doil C, Mailand N, Bekker-Jensen S, Menard P, Larsen DH, Pepperkok R et al. RNF168 binds and amplifies ubiquitin conjugates on damaged chromosomes to allow accumulation of repair proteins. Cell 2009; 136: 435–446. 19203579 10.1016/j.cell.2008.12.041

[bib26] Pierce AJ, Johnson RD, Thompson LH, Jasin M. XRCC3 promotes homology-directed repair of DNA damage in mammalian cells. Genes Dev 1999; 13: 2633–2638. 10541549 10.1101/gad.13.20.2633 PMC317094

[bib27] Gunn A, Stark JM. I-SceI-based assays to examine distinct repair outcomes of mammalian chromosomal double strand breaks. Methods Mol Biol 2012; 920: 379–391. 22941618 10.1007/978-1-61779-998-3_27

[bib28] Moore N, Houghton J, Lyle S. Slow-cycling therapy-resistant cancer cells. Stem Cells Dev 2012; 21: 1822–1830. 21973238 10.1089/scd.2011.0477 PMC3376467

[bib29] Henriksson S, Farnebo M. On the road with WRAP53beta: guardian of Cajal bodies and genome integrity. Front Genet 2015; 6: 91. 25852739 10.3389/fgene.2015.00091 PMC4371746

[bib30] Murga M, Jaco I, Fan Y, Soria R, Martinez-Pastor B, Cuadrado M et al. Global chromatin compaction limits the strength of the DNA damage response. J Cell Biol 2007; 178: 1101–1108. 17893239 10.1083/jcb.200704140 PMC2064646

[bib31] Mok MT, Cheng AS, Henderson BR. The ubiquitin ligases RNF8 and RNF168 display rapid but distinct dynamics at DNA repair foci in living cells. Int J Biochem Cell Biol 2014; 57: 27–34. 25304081 10.1016/j.biocel.2014.09.027

[bib32] Luijsterburg MS, Acs K, Ackermann L, Wiegant WW, Bekker-Jensen S, Larsen DH et al. A new non-catalytic role for ubiquitin ligase RNF8 in unfolding higher-order chromatin structure. EMBO J 2012; 31: 2511–2527. 22531782 10.1038/emboj.2012.104 PMC3365417

[bib33] Freund A, Zhong FL, Venteicher AS, Meng Z, Veenstra TD, Frydman J et al. Proteostatic control of telomerase function through TRiC-mediated folding of TCAB1. Cell 2014; 159: 1389–1403. 25467444 10.1016/j.cell.2014.10.059 PMC4329143

[bib34] Oren M, Rotter V. Introduction: p53 – the first twenty years. Cell Mol Life Sci 1999; 55: 9–11. 10065147 10.1007/s000180050265 PMC11146882

[bib35] Sasaki YF, Saga A, Akasaka M, Yoshida K, Nishidate E, Su YQ et al. *In vivo* genotoxicity of *ortho*-phenylphenol, biphenyl, and thiabendazole detected in multiple mouse organs by the alkaline single cell gel electrophoresis assay. Mutat Res 1997; 395: 189–198. 9465930 10.1016/s1383-5718(97)00168-x

